# Fabrication and Characterization of Polylactic Acid Electrospun Scaffolds Modified with Multi-Walled Carbon Nanotubes and Hydroxyapatite Nanoparticles

**DOI:** 10.3390/biomimetics5030043

**Published:** 2020-09-02

**Authors:** Athanasios Kotrotsos, Prokopis Yiallouros, Vassilis Kostopoulos

**Affiliations:** 1 Department of Mechanical Engineering and Aeronautics, Patras University Campus, University of Patras, GR-26504 Patras, Greece; akotrotso@mech.upatras.gr (A.K.); prokopiscy@gmail.com (P.Y.); 2 Foundation of Research and Technology, Institute of Chemical Engineering Sciences (FORTH/ICE-HT), GR-26504 Patras, Greece

**Keywords:** polylactic acid, electrospinning, scaffolds, contact angle, porosity, mechanical properties, MWCNTs, hydroxyapatite

## Abstract

The solution electrospinning process (SEP) is a cost-effective technique in which a wide range of polymeric materials can be electrospun. Electrospun materials can also be easily modified during the solution preparation process (prior SEP). Based on this, the aim of the current work is the fabrication and nanomodification of scaffolds using SEP, and the investigation of their porosity and physical and mechanical properties. In this study, polylactic acid (PLA) was selected for scaffold fabrication, and further modified with multi-walled carbon nanotubes (MWCNTs) and hydroxyapatite (HAP) nanoparticles. After fabrication, porosity calculation and physical and mechanical characterization for all scaffold types were conducted. More precisely, the morphology of the fibers (in terms of fiber diameter), the surface properties (in terms of contact angle) and the mechanical properties under the tensile mode of the fabricated scaffolds have been investigated and further compared against pristine PLA scaffolds (without nanofillers). Finally, the scaffold with the optimal properties was proposed as the candidate material for potential future cell culturing.

## 1. Introduction

The solution electrospinning process (SEP) has been characterized so far as a novel and cost-effective method for the fabrication of fibrous structures. This method allows the fabrication of fibers with diameters ranging from a few nanometers up to 1 mm. SEP has a lot of benefits, as it exhibits simplicity of use, adaptability and versatility [1]. A wide range of polymers can be electrospun, while the fabricated fibrous structures named scaffolds have shown to excellently mimic the extracellular matrix (ECM) environment to various degrees during the culture process of various cell types [2,3,4]. However, one of the most basic requirements for a scaffold’s functionality is sufficient mechanical properties (i.e., stiffness and strength), which further provide adequate stability. A scaffold not only provides an area for cell residence, but also maintains the mechanical stability at the defect point of the host human body [5]. Based on that, the mechanical properties of a scaffold are of great importance in the tissue engineering domain. In order for a well-structured scaffold to be effective, it must retain its structural integrity during handling and implantation at the defect site and provide sufficient biomechanical support during the regeneration process and the scaffold’s degradation [6].

A variety of polymers, either biodegradable or non-biodegradable, have been utilized so far for scaffold fabrication (i.e., delivery matrices), and the choice of the desirable polymer depends on each specific application [7,8,9]. Polylactic acid (PLA) has attracted the interest of various researchers over the past 40 years. PLA is polylactic acid (polylactide), which is a compostable thermoplastic aliphatic polyester, and is produced by a lactide ring opening polymerization process. It is also biodegradable, biocompatible, and has good mechanical properties. It can be easily dissolved in common solvents and decomposes over a reasonable period [10,11,12]. The decomposition products of PLA are charmless and fully metabolized by the human organism. A wide range of publications exist in the literature in which electrospun PLA is utilized as a tissue scaffold material [13,14,15,16,17,18,19,20,21], and it has been noted that by tuning the polymer concentration of the solution, scaffolds with different morphologies are obtained [15,21].

Scaffolds’ nanomodification by inorganic particles has been repeatedly reported in the literature. Noh et al. [22] fabricated PLA nanofibers modified with bioactive glass nanocomponents, which was proven to be useful for supporting a hard tissue regeneration matrix. Thermal treatments were also employed to further improve scaffolds’ performances. You et al. [23] enhanced scaffolds’ mechanical performances after heat treatment, as fibers were thermally bonded. Afifi et al. [24] also observed an analogous behavior. Lately, various researchers have focused on the nanomodification of electrospun fibers by carbon nanotubes (CNTs) and hydroxyapatite (HAP) for biomedical applications, including biosensors, drug delivery and the delivery of cell agents [25,26,27,28]. CNTs are long, thin graphene tubes that possesses unique mechanical and electrical properties that can be exploited to create biomimetic tailored scaffolds [29]. On the other hand, ceramic scaffolds composed of HAP have also been utilized so far. Modified scaffolds with HAP present structural similarities to the mineral phase of bone and are characterized as osteoconductive [28]. HAP is utilized as a bone substitute as it is stiffer and offers improved mechanical performance to a scaffold [30], but very often, scaffolds modified with HAP are fragile and have low porosity [31]. These scaffold types (with CNTs or HAP) can be easily fabricated by the SEP technique. 

McCullen et al. [32] developed a multi-walled carbon nanotube (MWCNT)-doped PLA scaffold intended for tissue engineering. By optimization, it was shown that the appropriate PLA concentration in a chloroform and dimethylformamide solvent combination was 20% *w*/*w*, while by the addition of the MWCNTs, the fiber diameter was reduced by 70%. Yang et al. [33] investigated the effect of PLA solution concentration, the solvent effect, and the CNT loadings on the final PLA fibrous composite structure. According to their experimental results, it was shown that, by using mixed solvents of chloroform/assistant solvent (*v*/*v* 3/1), better morphologies were achieved compared to using chloroform as a single solvent. On the other hand, by increasing CNT loadings, entangled bundles along the fiber axis were observed to have a misshapen morphology. Mackle et al. [29] also fabricated and characterized CNT-loaded electroactive PLA scaffolds. Morelli et al. [28] fabricated pristine PLA- and HAP-modified ones (with 20% and 50% *w*/*w*) for bone tissue engineering purposes. The obtained scaffolds presented different characteristics in terms of fiber diameter, porosity and mechanical properties. In addition, the differentiation of human mesenchymal stem cells from bone marrow in osteoblasts and the osteogenesis in the developed scaffold were investigated.

Herein, pristine PLA scaffolds and nanomodified ones, made by the SEP technique, have been successfully fabricated using a new solvent combination against other investigations. More precisely, pristine PLA scaffolds and nanomodified scaffolds with (a) HAP (1% and 2% *w*/*w*) and (b) MWCNTs (1% and 2% *w*/*w*) have been fabricated and further investigated. This work is an extension of and complements the works [27,30,34], in which scaffolds made by different biocompatible materials were fabricated and further investigated. The scope of this investigation was to fabricate and characterize PLA electrospun scaffolds (pristine and nanomodified) and study whether their physical and mechanical properties are improved/altered by nanomodification. Just after fabrication, scanning electron microscopy (SEM) and transmission electron microscopy (TEM) examinations were conducted in order to determine the mean fiber diameter, the morphology of the fabricated scaffolds, and the nanoparticle distribution through the fibers’ structure. In addition, the porosity value for all fabricated scaffolds was calculated. Mean fiber diameter and porosity values are of great interest, as they mainly affect the mechanical performance and further the cell activity (i.e., during incubation and proliferation) [35,36]. Furthermore, contact angle experiments were conducted to determine the hydrophilicity levels of the fabricated structures. Finally, the apparent mechanical properties under tensile loading were determined. Taking into consideration the experimental work conducted above, the fabricated structure that exhibited the best performance could be further utilized as a host scaffold structure for bone tissue regeneration purposes. 

## 2. Materials and Methods

### 2.1. Raw Materials

The utilized PLA material is a compostable thermoplastic aliphatic polyester polymer and it was developed by Innofil3D, Hauge, The Netherlands. PLA has a glass transition temperature (Tg) of about 50–60 °C and density of about 1240 kg/m^3^. Tetrahydrofuran (THF, inhibitor-free for HPLC ≥ 99%) and N, N Dimethylformamide (DMF, anhydrous ≥ 99.8%), which played the solvent role, were utilized and purchased by Sigma Aldrich, Saint Louis, MO, USA. Regarding the nanofillers, MWCNTs and a synthetic “needle-like” HAP were utilized. The obtained MWCNTs (identification code: NC7000) were supplied by Nanocyl, Sambreville, Belgium, and were produced via the Catalytic Chemical Vapor Deposition (CCVD) process. The typical MWCNTs diameter is between 10 and 20 nm and their aspect ratio spans were between 50 and 100. The synthetic “needle-like” HAP has an average length and thickness of 150 nm and 20 nm, respectively, a purity ≥ 97.5%, and it was purchased by Shanghai Xinglu Chemical Tech., Shanghai, China. In addition, HAP contains many minerals (i.e., Mg ≤ 1.8%, Na ≤ 0.2%, Fe ≤ 0.08% and Al ≤ 0.1%) according to the given specifications of the supplier. 

### 2.2. Solution Preparation and Solution Electrospinning Process (SEP)

The PLA material was dissolved into a mixture of THF/DMF solvents (80/20 *w*/*w*). Such a balance between the two solvents was proven after optimization to be necessary for obtaining dry fibrous structures. The utilized solvent combination is applied for the first time, as previous works that investigated PLA scaffolds chose different ones [13,14,15,16,17,18,19,20,21,22,23,28,29,32,33]. Then, solutions containing 20 wt. % PLA were prepared, leading to a final scaffold structure (after SEP) of (a) pure PLA (b) PLA with 1% HAP, (c) PLA with 2% HAP, (d) PLA with 1% MWCNTs and (d) PLA with 2% MWCNTs. All the PLA solutions were characterized by low viscosity and such concentrations are expected to enhance the mechanical performance of the final scaffolds and to obtain ultrafine fibers by avoiding electrospinnability problems during SEP. The electrospinnability problems occur due to the increase in the electrical conductivity value of the solution by introducing the conductive phase into it. 

The selected solutions were prepared by stirring for 24 h at room temperature (RT) to achieve homogeneity. Then, each solution type was loaded into a 3 mL syringe and it was directly electrospun onto a grounded aluminum foil-covered collector (15 × 15 cm^2^). The solution flow rate for all samples was fixed at 2.3 mL/h and the applied voltage was kept constant at 12 kV to achieve continuous jet formation. The distance between the tip of the nozzle and the collector was set to 14 cm and a metallic, 18 gauge (G18) hypodermic needle (with inner diameter equal to 0.84 mm) was used. The SEP was carried out at ambient conditions. For the needs of the present study, a lab-made SEP set-up was utilized, containing a stable flat square aluminum plate as collector.

### 2.3. Scanning Electron Microscopy (SEM), Transmission Electron Microscopy (TEM) and Micro-Structure Analysis

The morphology and micro-structure of the fabricated scaffolds were evaluated by using SEM and TEM microscopy. For the SEM, square strips with dimensions of 5 × 5 mm^2^ were machined and then sputter coated with gold for 30 s. Then, the samples were placed inside the field emission scanning electron microscopy instrument (FE-SEM, FEI InspectTM F50) by using a scanning electron (SE) detector. The FE-SEM instrument operated at 5 kV. On the other hand, circular carbon coated copper grids (200 mesh) with diameter 3.05 mm were utilized for the TEM characterization experiments. These grids were positioned carefully on the collector’s surface, so that the electrospun fibers could be deposited during the SEP. The TEM instrument model was the JEM-2100/ HR-TEM, constructed by the Japan electron optics laboratory (JEOL), and it operated at 200 kV.

The fiber diameter measurements were obtained by image processing with imageJ software (NIH, Bethesda, MD, USA). By using different thresholds, the SEM micrographs were converted to binary images. At least 150 fiber diameters were measured (50 measurements from three different samples’ images), while the average value and the standard deviation (SSD) were reported. In addition, the statistical test for the measured values of the fiber diameter was carried out by using the OriginPro with 95% confidence interval to determine the fiber diameter distribution, which best describes the experimental data.

### 2.4. Porosity Calculation

The porosity value of the scaffolds was determined by using the ratio of the measured mass of the sample to the mass of a fully dense sample of the same size, by measuring the sample’s dimensions (i.e., length, width and thickness). The thickness of all scaffolds was measured with a thickness gauge by applying constant force. The porosity was determined by using Equation (1):

(1) $$P=\frac{M_{1}-M_{2}}{M_{1}}\cdot 100\;\left(\%\right)\;$$

where P is the porosity, M_1_ is the mass of a fully dense sample and M_2_ is the mass of an electrospun scaffold. All the utilized samples had the same dimensions for comparison reasons.

### 2.5. Static Water Contact Angle Assay

Having the aim of evaluating the surface hydrophilicity of the fabricated electrospun scaffolds, static water contact angle experiments were conducted by using a contact angle goniometer apparatus (identification code: Kruss DSA100). The wetting characterization has a significant effect on bone mechanics because it constitutes an agent of biocompatibility. The utilized apparatus consists of a telescope equipped with a protractor to measure the angle of the tangent at the three-phase contact point of the static drop by using a camera together with a drop-shape analysis software. For the accurate wetting characterization of the PLA scaffolds, a 29 gauge (G29) stainless-steel needle (with an inner diameter of 0.184 mm) was controlled by a motor, so as to inspect the volume of the drop and to avoid unwanted vibration. The measurements were taken at t = 0 s after a single droplet of bi distilled water (2 μL) came into contact with the surface of the fibrous scaffolds. Four measurements were taken for each sample and the experiments were performed at RT conditions.

### 2.6. Uniaxial Tensile Tests

Five rectangular samples of 30 mm length and 10 mm width were punched out from each scaffold type. The thickness of each specimen was measured to be approximately 0.2 ± 0.01 mm by using a thickness gauge. For all samples, constant force was applied during the thickness measurements. Special care was also taken during the preparation to avoid severe damage to the samples. In addition, duct tape was carefully placed at each edge of the samples to improve the mounting of them on the metallic grips of the testing device. The uniaxial micro-tensile tests were performed on a minimat 2000 (rheometric scientific) tensile instrument with a 200 N load cell. During the experiments, the span length was set at 12 mm. All experiments were performed under RT conditions until sample’s failure at a cross head velocity of 5 mm/min. Prior to the testing, pre-tension was applied to all samples in order to ensure the extension and the receipt of load at the beginning of each experiment. The apparent mechanical properties of the scaffolds (apparent stress (*σ*) and strain (*ε*)) can be calculated at any time during the tensile experiments by using the following equations (Equations (2) and (3), respectively):

(2) $$\sigma =\frac{F}{A}$$

(3) $$\varepsilon =\frac{L-L_{o}}{L_{o}}$$

where *F* is the force, *A* is the cross-sectional area of the sample, *L* is the displacement and *L_o_* is the span length. The apparent mechanical properties of the scaffolds, ultimate tensile strength (*σ*_max_), young’s modulus (E) and elongation at break (*ε*_max_) were measured and further analyzed.

## 3. Results and Discussion

### 3.1. Structural and Morphological Analysis after Solution Electrospinning Process (SEP)

**Figure 1:**
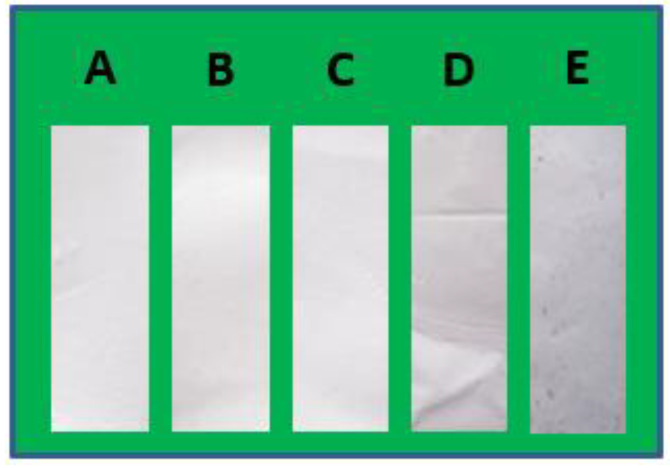
Illustration of the fabricated polylactic acid (PLA) scaffolds for the needs of the current investigation. (**A**) Pristine PLA, (**B**) modified PLA with 1% hydroxyapatite (HAP), (**C**) modified PLA with 2% HAP, (**D**) modified PLA with 1% multi-walled carbon nanotubes (MWCNTs) and (**E**) modified PLA with 2% MWCNTs.

The structure and the morphology of the fabricated PLA scaffolds (pristine and nanomodified scaffolds with HAP and MWCNTs) were investigated by using the SEM and the TEM microscopies. Figure 1 shows the fabricated scaffolds in their final form, which macroscopically are characterized as uniform and homogeneous. All the scaffold types have a characteristic white color apart from the samples containing MWCNTs, which appear to have light grey color (due to the black color of the MWCNTs). In the case of the modified PLA scaffolds with 2% MWCNTs, dark spots are evident on the picture (Figure 1E), which demonstrates the presence of agglomerates in the final scaffold. On the other hand, the samples containing HAP keep their initial color because the HAP appears to have the same one as the PLA fibers.

**Figure 2:**
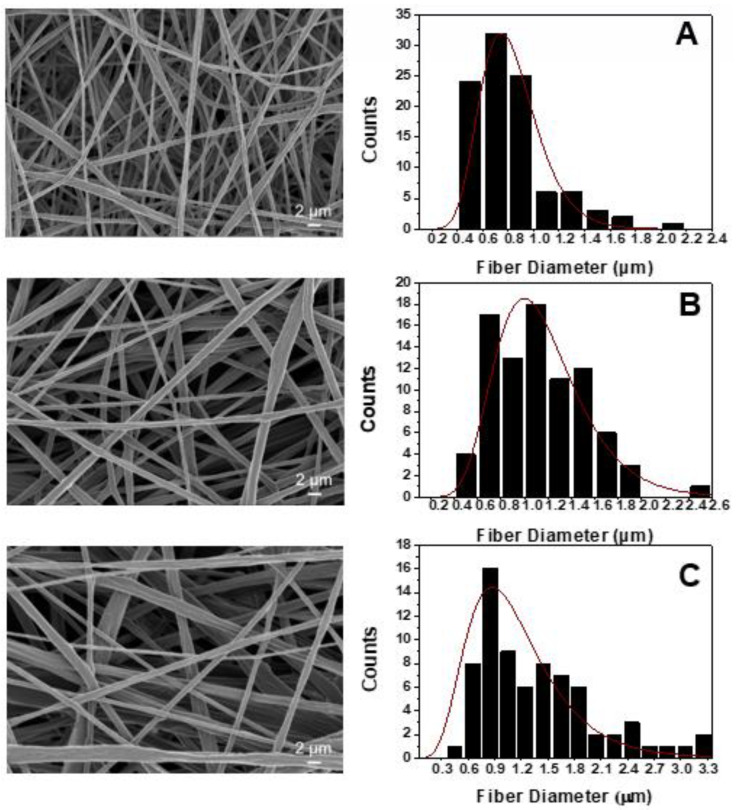

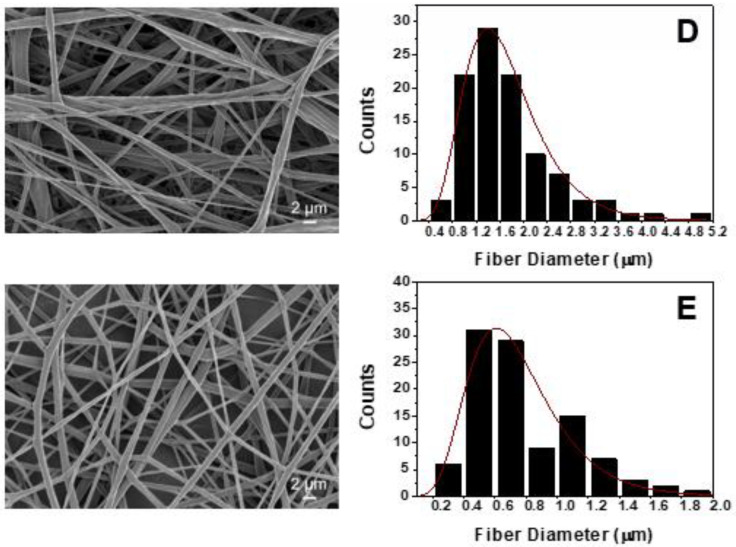
SEM images of (**A**) the pristine PLA, (**B**) the modified PLA with 1% HAP, (**C**) the modified PLA with 2% HAP (**D**) the modified PLA with 1% MWCNTs and (**E**) the modified PLA with 2% MWCNTs, together with histograms providing the fiber diameter distribution (red curves: fiber distribution curves).

**Figure 3:**
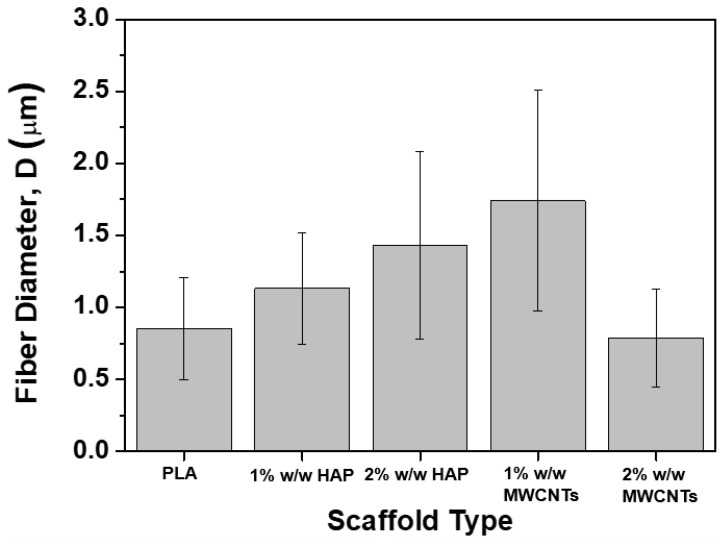
Bar chart diagram, providing the average fiber diameter values together with standard deviations (SSDs) for all scaffold types (pristine and nanomodified).

Figure 2 provides the SEM images that arose from each scaffold type together with the fiber diameter distribution histograms, according to specifications described in Section 2.3. Figure 2A–E corresponds to the pristine PLA (Figure 1A), to the 1% HAP modified (Figure 1B), to the 2% HAP modified (Figure 1C), to the 1% MWCNTs modified (Figure 1D) and to the 2% MWCNTs modified (Figure 1E), respectively. Based on the SEM images obtained for all material sets, cylindrical bead-free, non-woven and smooth fibers were fabricated for all cases, consisting of dense networks with no particular alignment (due to the SEP technique nature). The average fiber diameter values together with the standard deviations (SSDs) of them are summarized in Figure 3. According to the histograms of Figure 2 and the bar chart diagram of Figure 3, it was shown that nanomodification by HAP led to increased average fiber diameter. More precisely, the average fiber diameter was increased from 0.85 ± 0.35 μm for the nanofiller-free scaffolds to 1.13 ± 0.39 μm (33% increase) for scaffolds containing 1% w/w HAP, and to 1.43 ± 0.65 μm (68% increase) for scaffolds containing 2% *w*/*w* HAP. The current behavior observed (i.e., increases in the fiber diameter) is in line with the investigation of Morelli et al. [28]. On the other hand, the scaffolds containing 1% *w*/*w* MWCNTs exhibited higher average fiber diameter values, which was calculated to be more than doubled to 1.74 ± 0.76 μm (104% increase), while the samples containing 2% *w*/*w* MWCNTs exhibited slightly decreased values, by 8%, calculated to be close to 0.78 ± 0.34 μm. This behavior possibly occurred due to the increase in the electrical conductivity value of the solution, as the MWCNT quantity seems to have exceeded a threshold. Based on that, during the SEP, the average fiber diameter of the scaffold slightly decreased against the pristine ones. An analogous behavior was also observed in the investigation of Repanas et al. [34].

In the case of the scaffolds containing 2% *w*/*w* MWCNTs, the presence of MWCNT nanoparticles in the PLA solution seems to have led to an electrostatic charge build up during the SEP. As the jet exits the nozzle, it enhances the effect of the electric field in such a way as to obtain thinner fibers. Such an MWCNT quantity seems to have led to a lowering of the surface tension of the solution and resulted in an enhancement of the bending instability during the SEP. 

**Figure 4:**
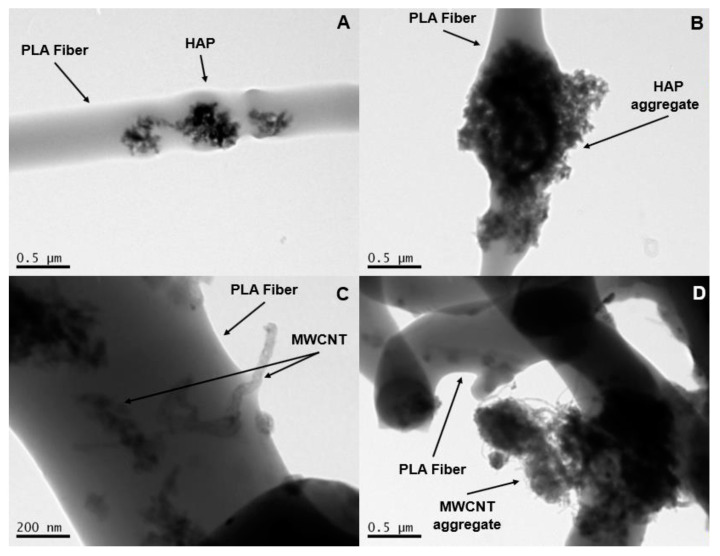
Transmission electron microscopy (TEM) images arose from the PLA scaffolds modified with (**A**) 1% *w*/*w* HAP, (**B**) 2% *w*/*w* HAP, (**C**) 1% *w*/*w* MWCNTs and (**D**) 2% *w*/*w* MWCNTs.

Having the aim to internally investigate the structure of the PLA fibers containing either HAP or MWCNTs, TEM microscopy examination was conducted according to specifications described in Section 2.3. Figure 4A–D corresponds to the scaffolds containing 1% *w*/*w* HAP (A), 2% *w*/*w* HAP (B), 1% *w*/*w* MWCNTs (C) and 2% *w*/*w* MWCNTs (D), respectively. According to the obtained images, it was shown that in both cases, by increasing the content of the nanoparticles, aggregates appear more and more with no particular nanofiller alignment (especially at higher concentrations). The best nanofiller distribution seems to have been achieved for scaffolds containing 1% *w*/*w* MWCNTs, which is expecting to further improve the mechanical properties of the scaffold. 

### 3.2. Porosity

**Figure 5:**
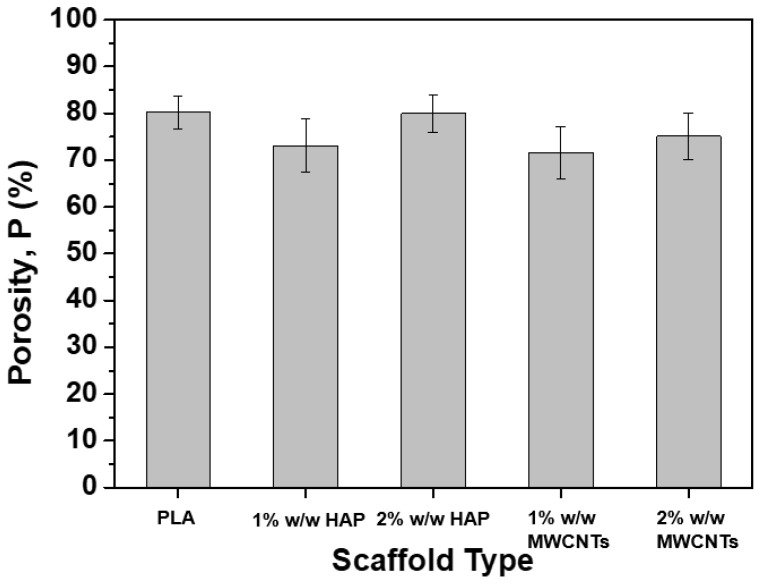
Bar chart diagram, providing the porosity (*P*) values for all the scaffold types (pristine and nanomodified).

Scaffold nanomodification by HAP or MWCNTs in various nanofiller concentrations (1% and 2% *w*/*w*) is also expected to have an impact on the porosity value of the final sample. Based on that, the porosity value for all the scaffold types was calculated according to specifications described in Section 2.4, and is provided in the bar chart diagram of Figure 5. According to this figure, the general trend for all the scaffold types is the reduction of the porosity value by nanomodification. More precisely, the porosity mean value was reduced by 9%, 0.3%, 10.7% and 6.5% for the samples containing 1% *w*/*w* HAP, 2% *w*/*w* HAP, 1% *w*/*w* MWCNTs and 2% *w*/*w* MWCNTs, respectively. The current findings are in line with the investigations of Kostopoulos et al. [27.30], in which the scaffold nanomodification mainly contributed to a slight reduction of the porosity value compared to the pristine scaffold. However, based on Chen et al.’s [37] investigation, the current porosity values are expected to support the interconnectivity, the interaction and the proliferation procedure of the cells.

### 3.3. Static Water Contact Angle Assay

**Figure 6:**
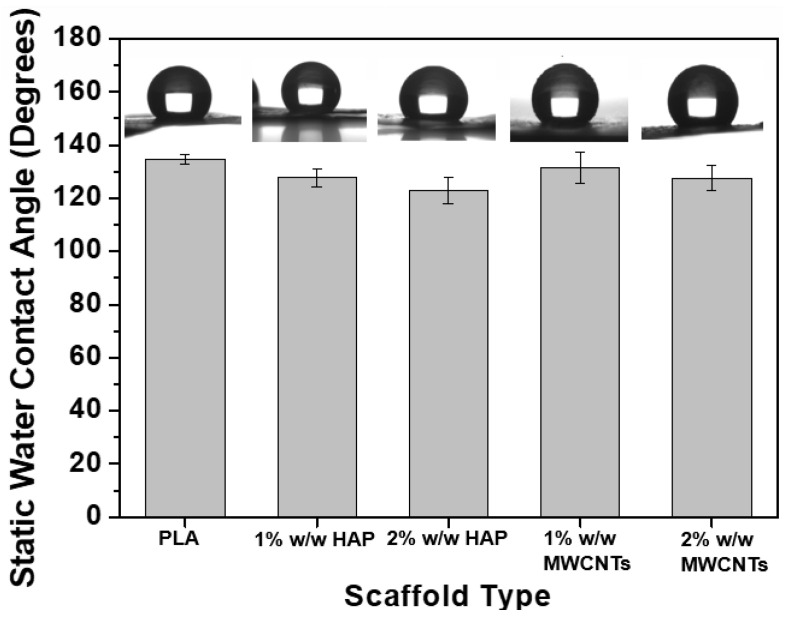
Bar chart diagram, providing the average contact angle value of the fabricated PLA scaffolds together with SSD.

The current section investigates the effect of the nanofiller inclusion and their concentration on the hydrophilicity levels of the fabricated PLA scaffolds. In this regard, static water contact angle assay experiments were conducted according to specifications described in the Section 2.5. The bar chart diagram of Figure 6 provides the static contact angles for all the material sets.

According to experimental results, it was shown that all the scaffold types presented static contact angle values above 90°. Such values indicate the hydrophobic nature of all the scaffolds, but the nanomodification in its turn resulted in less hydrophobic materials as compared to pristine PLA scaffolds. Both HAP and MWCNT nanoparticles slightly improved the hydrophilicity levels of the PLA scaffold. Similar findings were also observed in Kostopoulos et al.’s [27,30] investigations. Based on the experimental observations, it was shown that the scaffolds containing 1% w/w HAP exhibited slightly decreased contact angle values, by 5.2% (from 134.76 ± 1.84° to 127.70 ± 10.54°), while for the samples containing 2% w/w HAP the contact angle value was slightly reduced by 8.8% (from 134.76 ± 1.84° to 122.90 ± 4.95°). In the presence of MWCNTs, the same behavior was also observed. Scaffolds containing 1% and 2% *w*/*w* MWCNTs exhibited slightly reduced contact angle values, by 2.4% (from 134.76 ± 1.84° to 131.50 ± 5.94°) and 5.3% (from 134.76 ± 1.84° to 127.56 ± 4.72°), respectively.

### 3.4. Mechanical Properties Under Tensile Mode

In this section, the effects of the nanofiller type and the concentration on the apparent mechanical properties of the fabricated scaffolds are investigated by conducting uniaxial tensile tests, according to the specifications of Section 2.6 The rectangular samples that were machined from each scaffold type were carefully mounted on the testing cards and gripped to the tensile apparatus. 

**Figure 7:**
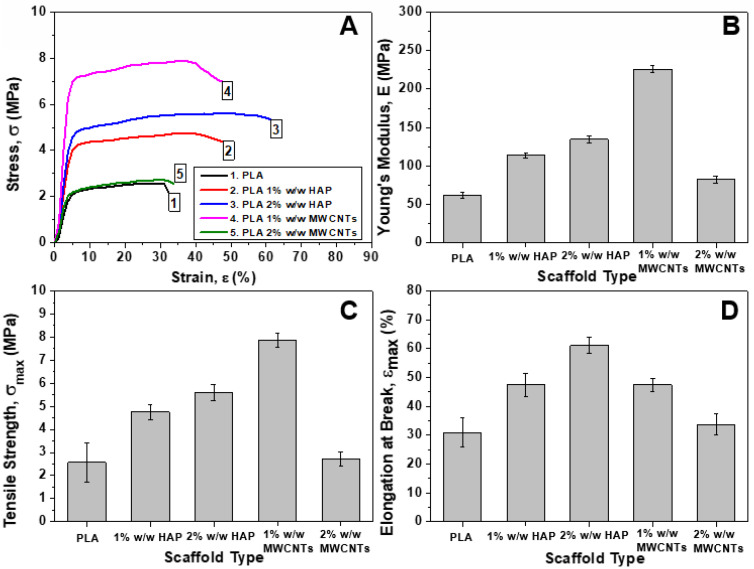
(**A**) Representative stress (*σ*) vs. strain (*ε*) % curves for the pristine PLA and nano-modified scaffolds (with 1% and 2% HAP, and 1% and 2% MWCNTs). Bar chart diagrams providing the tensile properties of PLA scaffolds that arose from uniaxial tensile tests; (**B**) Young’s Modulus (*E*), (**C**) Tensile strength (*σ_max_*), (**D**) Elongation at break (*ε_max_*) %. For all the tensile properties SSD values are provided.

In Figure 7A, representative stress (*σ*) vs. strain (*ε*) % curves for the pristine PLA and nanomodified scaffolds with HAP and MWCNTs in various concentrations are illustrated. For each scaffold type, the apparent *E*, *σ_max_* and *ε_max_* (%) were calculated and are provided in the bar charts of Figure 7B–D. The general trend does not present any dramatic difference for all the scaffold types (pristine PLA and nanomodified). The applied stress increases linearly up to a certain extent, as the initially linear *σ*-*ε* (%) relation is followed by a distinguishable deviation from linearity. Much later, a plateau is formed, followed by a load drop up to the fracture of the samples. The bar chart of Figure 7B presents average values of the *E* value for all the scaffold types. These values were calculated through linear regression analysis of the the initial linear parts of the *σ*-*ε* curves. The average *E* value of the pristine PLA scaffolds was calculated to be 62.4 ± 3.9 MPa. The samples containing 1% and 2% w/w HAP exhibited improved *E* values by 82% (114 ± 3.4 MPa) and 115% (134.6 ± 4.5 MPa), respectively. In the case of MWCNT-modified scaffolds, the *E* values were enhanced by 262% (226 ± 7.7 MPa) and 31.7% (82.2 ± 4.5 MPa) for the scaffolds containing 1% *w*/*w* and 2% *w*/*w* MWCNTs, respectively. In this regard, the *σ_max_* values were also improved by 84.48% (from 2.6 ± 0.9 MPa to 4.8 ± 0.3 MPa) and 117.9% (from 2.6 ± 0.9 MPa to 5.6 ± 0.4 MPa) for the scaffolds containing 1% w/w and 2% *w*/*w* HAP, respectively. The samples containing 1% and 2% MWCNTs exhibited improved *σ_max_* values by 206.6% (from 2.6 ± 0.9 MPa to 7.9 ± 0.3 MPa) and 6.2% (from 2.6 ± 0.9 MPa to 2.7 ± 0.3 MPa), respectively. Finally, the *ε_max_* (%) value was significantly improved for all the scaffold types, ranging from 30% to 60%. 

**Figure 8:**
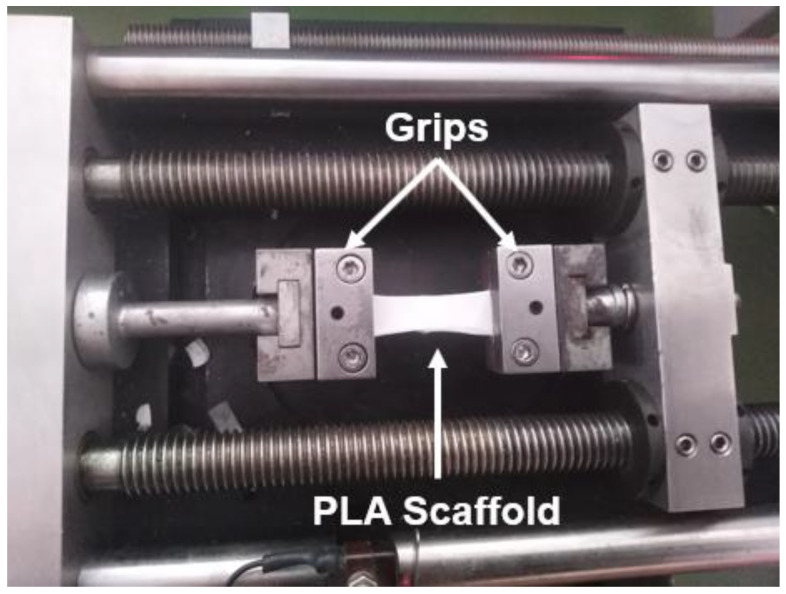
Snapshot taken during the uniaxial tensile tests of the PLA scaffolds.

According to the experimental results provided and discussed above, it was shown that for all the scaffold types the mechanical properties under tensile mode were improved with the scaffolds containing 1% *w*/*w* MWCNTs, and exhibit the best mechanical performance. According to the TEM image of Figure 4C, in this type of sample a good dispersion of the MWCNTs seems to have been achieved. In addition, these scaffolds presented a higher average diameter value and a relatively low porosity value, which in its turn led to significantly improved tensile properties. For the samples containing 2% *w*/*w* MWCNTs, the strong presence of the aggregates (see Figure 4D), most of which were found outside the fiber structure, resulted in weaker scaffolds with significantly reduced tensile properties as compared to the 1% *w*/*w* MWCNT-modified scaffolds, but they exhibited slightly improved behavior if compared to pristine PLA scaffolds. Furthermore, the pristine PLA and 1% *w*/*w* MWCNT-containing scaffolds presented comparable average fiber diameter values. In the case of scaffolds modified with HAP, it was shown that by increasing the HAP concentration the tensile properties were enhanced even if the aggregate presence is made stronger by increasing the HAP concentration (see in Figure 4A,B). Similar findings were also observed in Kostopoulos et al.’s [30] and Gkermpoura et al.’s [38] investigations, in which various scaffolds were nanomodified with various nanoparticles. In Figure 8, a snapshot of the tensile testing is illustrated, indicated the ductile nature of the precise PLA scaffolds. 

## 4. Conclusions

The current study investigated the effect of nanofiller type and concentration on the PLA scaffold’s structural, physical and mechanical properties. More precisely, HAP and MWCNTs were employed to modify pristine PLA scaffolds at various concentrations (i.e., 1% and 2% *w*/*w*). After the fabrication and according to the SEM examinations, it was shown that all the scaffold types presented acceptable structures for use in supporting cell cultures in future. Beadless cylindrical structures consisting of non-woven dense networks were fabricated with the 1% w/w MWCNT-containing scaffolds to exhibit the highest average fiber diameter value and lowest porosity (compared to the pristine scaffolds). For all the scaffold types it was shown that by increasing the nanofiller content, the nanofiller aggregates were more and more detectable according to the obtained TEM images. In general, the average porosities and the contact angle values were reduced by the nanomodification in all the scaffold types. Based on these, the obtained scaffolds were less hydrophobic against pristine scaffolds (the contact angle values are still higher than 90°), while the mechanical properties were improved. According to the tensile experiments conducted, all the scaffolds presented improved mechanical properties (mainly due to the nanofiller presence and the lower porosities) with 1% *w*/*w* MWCNT-containing scaffolds and exhibited the best mechanical performance. This scaffold seems to be the most promising one compared to others as regards supporting cell cultures in future. Finally, this type of scaffold could also be utilized for other potential applications, such as drug delivery, filtration, batteries and composite materials.
